# Effects of aerobic exercise training on the risk factors for liver diseases in elderly women with obesity and impaired fasting glucose: A pilot study

**DOI:** 10.20463/jenb.2019.0004

**Published:** 2019-03-31

**Authors:** Jae Ho Park, Hee-jae Kim, Aleum Han, Deuk-mo Kang, Sok Park

**Affiliations:** 1 Institute of Sports Medicine & Nutrition, Kwangwoon University, Seoul Republic of Korea; 2 Institute of Sports Science, Seoul National University, Seoul Republic of Korea; 3 Department of Physical Education, Sejong University, Seoul Republic of Korea

**Keywords:** Obesity, Impaired fasting glucose, Liver disease, Aerobic exercise

## Abstract

**[Purpose]:**

In the present pilot study, we aimed to investigate the effects of the Silverrobics exercise program, which is similar to aerobic dance, on the factors related to glucose metabolism and liver enzymes.

**[Methods]:**

Eight elderly women with obesity and impaired fasting glucose participated in the Silverrobics exercise program (60 minutes per session for five times a week for 8 weeks). The program was conducted at 50–60% of the heart rate reserve at 1 to 2 weeks and at 60–80% of the heart rate reserve at 3 to 8 weeks. To verify the effect of this 8-week exercise program on glucose metabolism and liver enzymes, blood analysis at pre- and post-training was performed.

**[Results]:**

After the Silverrobics exercise program, there were significant decreases in the glucose (p<0.05), glycated hemoglobin A1c (p<0.05), 1,5-anhydroglucitol (p<0.05), and insulin levels (p<0.01) and homeostatic model assessment of insulin resistance score (p<0.05). However, there were no significant effects on the liver enzymes, except for alkaline phosphatase. The alkaline phosphatase level increased after the Silverrobics exercise program (p<0.05).

**[Conclusion]:**

Although the Silverrobics exercise program had no beneficial effects on the liver enzymes, it may play an important role in preventing liver diseases considering the effects on glucose metabolism.

## INTRODUCTION

Obesity has become a global public health problem. According to the World Health Organization, approximately 39% of adults are overweight, and 13% are obese^1^. Although there is a great global obesity pandemic, the prevalence of obesity is greater in women than in men in most populations^2^^-^^5^. There is accumulating evidence that the prevalence of sarcopenic obesity in elderly women is higher than that in elderly men; thus, early management of body composition with adequate lifestyle changes is more important for women than for men^6^. Epidemiological evidence suggests that menopausal transition is an important factor for increasing body weight and fat mass^7^.

Liver diseases, such as nonalcoholic fatty liver disease (NAFLD), are strongly related to obesity^8^^-^^11^. Although only 18% of individuals with a normal body mass index (BMI) are patients with NAFLD, up to 70% of patients with NAFLD are obese^12^. The resulting obesity epidemic has made liver diseases, such as NAFLD, become one of the common diseases worldwide^13^^,^^14^. NAFLD should be prevented because it can progress to nonalcoholic steatohepatitis (NASH), which can consequently increase the risk of cirrhosis or even hepatocellular carcinoma (HCC)^15^^,^^16^. NAFLD is marked by upregulated liver enzymes, such as aspartate aminotransferase (AST) and alanine aminotransferase (ALT)^17^. Further, there are other enzymes related to liver damage and function, such as alkaline phosphatase (ALP) and albumin^18^. Moreover, the AST/ALT ratio is a critical risk factor for predicting liver damage because the levels of AST increase more than those of ALT when hepatocellular death increases^19^.

Obesity is also one of the major causes of insulin resistance (IR). Several inflammatory cytokines from fat tissues have been suggested as a major factor inducing IR. Inflammatory cytokines, such as tumor necrosis factor-α and interleukin-6, cause IR; thus, overexpression of these cytokines increases the prevalence of type 2 diabetes and metabolic syndrome^20^^,^^21^. Importantly, IR and obesity have been identified as critical risk factors for liver diseases, such as NAFLD and even HCC^22^^,^^23^. Further, impairment of the hepatic insulin signaling pathway is closely related to an increment in hepatocyte apoptosis in patients with liver diseases^24^. Moreover, high levels of glucose [i.e., impaired fasting glucose (IFG)] are closely associated with hepatic fat and hepatocyte apoptosis^25^^,^^26^. Therefore, it could be concluded that impaired glucose metabolism in obesity has clinical implications for liver diseases.

Exercise has been proven to be a good strategy for treating obesity and IFG. Considering the relationship between obesity and liver diseases explained above, it is likely that exercise could have beneficial effects on liver diseases. Exercise has been reported to affect NAFLD treatment positively via improvement of insulin sensitivity and reduction of body weight^27^. However, the role of exercise in treating NAFLD has not been completely identified, and what exercise regimen is the most beneficial for liver diseases is unknown^14^^,^^28^. A few studies have investigated the effect of exercise on both glucose metabolism and liver enzymes. A previous study reported significant reductions in the AST and ALT levels, as well as the value of IR, in Iranian men (age, 32–54 years) with NAFLD who adhered to an 8-week aerobic exercise or resistance exercise program^28^. These results demonstrate that both aerobic exercise and resistance exercise can alleviate the risk factors of liver diseases. There were also significant decreases in the AST and ALT levels in women with obesity (age, 18–65 years) who engaged in a 3-month aerobic exercise with resistance exercise^29^. These results showed that exercise can have beneficial effects on the risk factors of liver diseases in adult women (age, <65 years). However, the previous authors could not assess factors related to glucose metabolism. Considering the value of the IR as a risk factor of liver diseases, further studies are required for verification. To the best of our knowledge, there are no data on the effects of aerobic exercise on both liver enzymes and glucose metabolism in elderly Korean women with obesity and IFG. Therefore, this study was conducted.

The purpose of this pilot study was to investigate whether regular aerobic exercises in elderly women with obesity and IFG have beneficial effects on both glucose metabolism and liver enzymes. We expect the preliminary results to offer relevant information for designing further studies and facilitating planning of the exercise program and management of participants. To verify this, we hypothesized that regular aerobic exercises in elderly women with obesity and IFG could enhance glucose metabolism, resulting in the reduction of the risk factors for liver diseases.

## METHODS

### Participants

Participants were openly recruited through advertising in the community and personal contact. We sought elderly women aged 65 to 70 years with obesity and IFG who had no medical history that could prevent them from attending an 8-week exercise program. The exclusion criteria included current usage of medications that can affect glucose metabolism and liver enzymes. According to the American Diabetes Association, IFG was defined as fasting glucose levels between 100 and 125 mg/dL^30^. Obesity was defined in accordance with the criteria used in the Asian and Pacific regions (BMI, ≥25 kg/m^2^)^31^. All volunteers had obesity and IFG. All participants provided written informed consent after explanation of the study and possible adverse effects. The anthropometric characteristics of the participants are shown in Table 2 .

### Procedures

Approximately 2 weeks prior to the baseline measurements, the participants visited the laboratory for familiarization with the pre-test. They were required to avoid performing any vigorous physical activity, drinking, and smoking 24 hours prior to the study. Further, the participants were recommended to get enough sleep. To verify the effect of the 8-week exercise on glucose metabolism and liver enzymes, blood analysis at pre- and post-training was performed. The participants underwent aerobic exercise training during the 8-week intervention period.

### Aerobic exercise training

All participants started the aerobic exercise following the baseline measurements. The aerobic exercise program used in this study was the Silverrobics exercise program (SEP), which is similar to aerobic dance. The SEP is a modified form of aerobic dance for elderly individuals. During the 8-week training period, all subjects participated in the SEP for 60 minutes per session (including warm-up and cool-down) five times a week under the instruction of an SEP expert and supervision of an exercise physiologist. The SEP consists of three phases: (1) warm-up, (2) main exercise, and (3) cool-down. At the beginning of the SEP session, the participants conducted 10 minutes of warm-up, including stand stretch and rhythmic step stretch. The warm-up was followed by the main exercise. The main exercise of the SEP consists of aerobic dance performed on music for 30 to 40 minutes. This phase was conducted at 50–60% of the heart rate reserve (HRR) and at 10–12 of the rating of perceived exertion (RPE; Borg scale) at 1 to 2 weeks. Thereafter, the intensity was progressively increased at 60–80% of the HRR and 10–16 of the RPE at 3 to 8 weeks. The target heart rate (THR) was calculated using the following formula: [THR = (maximal heart rate – resting heart rate) × target intensity (%) + resting heart rate]. The maximal heart rate was calculated using the following formula: (maximal heart rate = 220 – age). To measure the THR, each participant underwent heart rate monitoring using a polar heart rate monitor (Polar, Finland). Lastly, the participants performed cool-down, including light resistance exercise using a dumbbell or an elastic band and supine stretch, to allow their heart rate to drop slowly. The details of the SEP are shown in Table 1.

**Table 1:** Silverrobics exercise program

Phase		Exercise mode (HR)	Exercise periods					
			1-2 weeks	Intensity	3-5 weeks	Intensity	6-8 weeks	Intensity
Warm- up	Warm-up Hi- Warm-up	Stand Stretch (110-120)	5 min	HRR 50% -60%	5 min	HRR 50% -60%	5 min	HRR 50% -60%
		Step Stretch (120-130)	5 min	HRR 50% -60%	5 min	HRR 60% -70%	5 min	HRR 60% -70%
Main exercise		Aerobic dance (120-140)	20 min	HRR 50% -60%	20 min	HRR 60% -80%	20 min	HRR 60% -80%
		Aerobic step (120-130)	20 min	HRR 50% -60%	20 min	HRR 50% -60%	20 min	HRR 50% -60%
Cool-down	Light resistance exercise	Dumbbell Ceraband (100-120)	5 min	HRR 50% -60%	5 min	HRR 60% -70%	5 min	HRR 60% -70%
	Supine Stretch	Relax stretch (60-100)	5 min	HRR 40% -50%	5 min	HRR 40% -50%	5 min	HRR 40% -50%

HR: heart rate; HRR: heart rate reserve

### Outcome measures

#### Anthropometric measures

Anthropometric data included body weight and height presented to the nearest 0.01 kg and 0.01 cm, respectively. BMI was also calculated as body weight in kilograms divided by body height in meters squared (kg/m^2^).

#### Blood analysis

For determination of the factors related to glucose metabolism and liver enzymes, venous blood samples were obtained at pre- and post-training. Samplings at both pre- and post-training were performed at 10 am. Further, all participants were required to fast for 9 hours before extracting their blood sample. Approximately 10 mL of blood was extracted from the median antecubital vein and stored in an EDTA tube. The plasma was centrifuged for 15 minutes at 3,000 rpm at 4°C. Thereafter, the samples were stored frozen for subsequent analysis.

#### Serum glucose level and IR score

The serum glucose, insulin, glycated hemoglobin A1c (HbA1c), and 1,5-anhydroglucitol (1,5-AG) levels were measured via an absorptiometric analysis at 450 nm using an ELISA kit (MyBioSource, San Diego, CA, USA). To verify the changes in glucose metabolism, we additionally selected the HbA1c and 1,5-AG levels as glycemic markers. HbA1c is used as a marker to measure the mean glucose levels over a 3-month period, while 1,5-AG is a validated marker to evaluate short-term glycemic control^32^. Further, the homeostatic model assessment of insulin resistance (HOMA-IR) score, which is an indicator of IR, was calculated using the following formula^33^.

$$\text{HOMA-IR=}\left[\text{Insulin}\left(\text{μU/ml}\right)\text{×Glucose}\left(\text{mmol/L}\right)\right]\text{/22.5}$$

#### Biomarkers of liver injury

The levels of AST, ALT, and ALP, which are liver enzymes, and that of albumin, which is a protein, were measured using a biochemical analyzer (Hitachi, Tokyo, Japan). All of these are affected by liver damage. The AST, ALT, and ALP levels are elevated when the liver is injured, while lower than normal levels of albumin might indicate liver damage^34^. Moreover, the AST/ALT ratio is an important risk factor for predicting liver damage because the levels of AST increase more than those of ALT when hepatocellular death increases^19^.

### Statistical analysis

All data were analyzed using the SPSS software version 25.0 (SPSS Inc., USA). Data were presented as means ± standard deviations. To evaluate the changes in the factors related to glucose metabolism and liver function between pre- and post-training, the paired t-test was used. Statistical significance was set at p<0.05.

## RESULTS

### Serum glucose level

Table 3 demonstrates the effects of the 8-week SEP on the factors related to the serum glucose level. There were significant decreases in all variables. The reductions in the serum glucose level (from 112.50±20.00 to 94.50±9.43 mg/dL, p<0.05), HbA1c level (from 5.98±0.24 to 5.53±0.09%, p<0.05), and 1,5-AG level (from 7.26±0.31 to 6.57±0.19 μg/mL, p<0.05) were significant.

### Serum insulin level and HOMA-IR score

Table 4 demonstrates the effects of the 8-week SEP on the serum insulin level and HOMA-IR score. There were significant reductions in both variables. The decreases in the insulin level (from 17.31±7.18 to 8.07±3.94 μU/mL, p<0.01) and HOMA-IR score (from 5.57±3.25 to 2.36±1.39, p<0.05) were significant.

### Biomarkers of liver injury

Table 5 demonstrates the effects of the 8-week SEP on the factors related to liver dysfunction. There were no significant decreases in the AST, ALT, and albumin levels and AST/ALT ratio. The increase in the ALP level (from 54.75±9.72 to 55.63±10.01 U/L) was significant (p<0.05).

**Table 2:** Anthropometric characteristics of the participants (n=8)

Age (year)	Height (cm)	Weight (kg)	BMI (kg/m^2^)	Glucose level (mg/dL)
66.60±2.87	155.7±3.77	63.37±4.20	26.29±1.82	112.50±20.00

BMI: body mass index

**Table 3:** Glucose level before and after the training

	Glucose level (mg/dL)	HbA1c level (%)	1,5-AG level (μg/mL)
Pre-training	112.50±20.00	5.98±0.24	7.26±0.31
Post-training	94.50±9.43	5.53±0.09	6.57±0.19
p-value	<0.05	<0.05	<0.05

HbA1c: glycated hemoglobin A1c; 1,5-AG: 1,5-anhydroglucitol

**Table 4:** Insulin resistance before and after the training

	Insulin level (μU/mL)	HOMA-IR score
Pre-training	17.31±7.18	5.57±3.25
Post-training	8.07±3.94	2.36±1.39
p-value	<0.01	<0.05

HOMA-IR: homeostatic model assessment of insulin resistance

**Table 5:** Biomarkers of liver injury before and after the training

	AST level (U/L)	ALT level (U/L)	AST /ALT ratio	ALP level (U/L)	Albumin level (g/dL)
Pre- training	22.38±5.58	12.75±3.01	1.77±0.25	54.75±9.72	4.54±0.19
Post- training	22.13±5.79	11.50±3.89	2.02±0.57	55.63±10.01	4.53±0.20
p-value	0.516	0.250	0.208	<0.05	0.351

AST: aspartate aminotransferase; ALT: alanine aminotransferase; ALP: alkaline phosphatase

## DISCUSSION

The aim of this study was to investigate whether regular aerobic exercise in elderly women with obesity and IFG has a beneficial effect on both glucose metabolism and liver enzymes. Based on the experimental results, all variables related to glucose metabolism significantly decreased. However, in contrast to our expectations, the increase in the ALP level was significant. In summary, there were no differences in the training effects on the liver enzymes, while all factors related to glucose metabolism improved after the 8-week SEP.

There have been consistent efforts to investigate the effects of exercise on the risk factors for liver diseases. However, to the best of our knowledge, only a few studies have investigated the effects of the SEP on both glucose metabolism and liver function. Most studies on the risk factors for liver diseases have focused on the different effects between aerobic exercise and resistance exercise or the effects of combinations of exercise and dietary interventions. These previous studies have shown that there is no significant difference in the effects on the risk factors for liver diseases between these two exercise regimens^28^^,^^35^^-^^37^. Further, other studies showed that the combination of exercise and diet can have positive effects on glucose metabolism^38^, liver damage^39^^,^^40^, or both^41^^,^^42^. However, these studies could not determine whether these beneficial effects were induced by exercise or diet. Considering that only a few studies have investigated the effect of aerobic exercise training on liver disease risk factors associated with glucose metabolism and liver function at the same time^43^^,^^44^, further experiments applying various types of exercise are required to determine the best type of exercise for the prevention of liver diseases.

In this study, there were significant differences in the factors associated with glucose metabolism between pre- and post-training. This indicates that the SEP can have a beneficial effect on glucose metabolism. The participants’ glucose levels improved to a normal level (from 112.50±20.00 to 94.50±9.43 mg/dL). Improving glucose metabolism is one of the mechanisms suggested to prevent liver diseases. Lipolysis tends to increase in obesity; the release of excess free fatty acids (FFAs) into hepatocytes is augmented^45^. FFAs absorbed by hepatocytes are attached to coenzyme A (CoA) as fatty acyl-CoAs to shape hepatic triglycerides; this may induce a decrease in the insulin-induced glucose uptake^46^. However, fatty acyl-CoAs are not formed when the levels of FFAs are high; thus, this can stimulate intracellular inflammation and IR^47^. IR and hyperglycemia arising from this pathological state can promote liver apoptosis and hepatic steatosis^24^^,^^45^. Therefore, the results related to glucose metabolism may imply that the SEP may play a role in preventing obesity from progressing to liver diseases.

There were no significant differences in the factors related to liver injury between pre- and post-training. This indicates that the 8-week SEP had no beneficial effects on those variables. The reason why the factors, including the AST, ALT, and albumin levels and AST/ALT ratio, did not improve after the SEP might be that all participants already had normal levels before training. The participants had no NAFLD nor NASH; thus, their liver enzyme levels were normal even before the intervention. According to a meta-analysis of randomized trials, exercise cannot have an effect on liver enzymes, such as ALT, because the subjects already had normal levels^48^. Contrary to our results, exercise training reduced the ALT and AST levels in several trials of patients with liver diseases^17^^,^^28^^,^^49^. In contrast to our expectations, the ALP level increased after the 8-week SEP. There was a small increment in the ALP level, which was within the normal range (from 54.75±9.72 to 55.63±10.01 U/L). A previous study has reported a similar extent of increment in the ALP level after a 12-week endurance training (from 74.53±19.21 to 75.47±19.26 U/L)^29^. The serum ALP level also correlated positively with the visceral fat mass in middle-aged Koreans^50^. Thus, to determine the reason why the ALP levels increased after training, further studies measuring visceral fat between pre- and post-training should be conducted.

Our study has some limitations. First, our study was a pilot study including only one group; thus, we cannot ensure that the results were completely attributable to the exercise program. Therefore, further studies including a control group are required for verification of the interaction effects. Second, the sample size of the subjects was small. Further studies with larger sample sizes are necessary to augment the external validity of the present results. Lastly, the participants of our study were healthy individuals; thus, the application of our results to other medical conditions may be limited. Further studies on the effects of the SEP on various medical conditions are necessary to apply the SEP to various fields.

## CONCLUSION

Regular aerobic exercises (i.e., SEP) may improve the glucose metabolism of elderly women with obesity and IFG. Considering the factors associated with glucose metabolism, the SEP may play an important role in preventing liver diseases.
